# Explainable machine learning model and nomogram for predicting the efficacy of Traditional Chinese Medicine in treating Long COVID: a retrospective study

**DOI:** 10.3389/fmed.2025.1529993

**Published:** 2025-03-13

**Authors:** Jisheng Zhang, Yang Chen, Aijun Zhang, Yi Yang, Liqian Ma, Hangqi Meng, Jintao Wu, Kean Zhu, Jiangsong Zhang, Ke Lin, Xianming Lin

**Affiliations:** ^1^The Third Affiliated Hospital of Zhejiang Chinese Medical University, Hangzhou, China; ^2^Jiaxing First People's Hospital, Jiaxing, China; ^3^Haining People's Hospital, Jiaxing, China; ^4^The First Clinical Medical College of Zhejiang Chinese Medicine University, Hangzhou, China

**Keywords:** machine learning, SHapley Additive exPlanations, nomogram, Traditional Chinese Medicine, Long COVID, efficacy

## Abstract

**Introduction:**

Long COVID significantly affects patients' quality of life, yet no standardized treatment has been established. Traditional Chinese Medicine (TCM) presents a promising potential approach with targeted therapeutic strategies. This study aims to develop an explainable machine learning (ML) model and nomogram to identify Long COVID patients who may benefit from TCM, enhancing clinical decision-making.

**Methods:**

We analyzed data from 1,331 Long COVID patients treated with TCM between December 2022 and February 2024 at three hospitals in Zhejiang, China. Effectiveness was defined as improvement in two or more symptoms or a minimum 2-point increase in the Traditional Chinese Medicine Syndrome Score (TCMSS). Data included 11 patient and disease characteristics, 18 clinical symptoms and syndrome scores, and 12 auxiliary examination indicators. The least absolute shrinkage and selection operator (LASSO) method identified features linked to TCM efficacy. Data from 1,204 patients served as the training set, while 127 patients formed the testing set.

**Results:**

We employed five ML algorithms: Support Vector Machine (SVM), Random Forest (RF), K-Nearest Neighbors (KNN), Extreme Gradient Boosting (XGBoost), and Neural Network (NN). The XGBoost model achieved an Area Under the Curve (AUC) of 0.9957 and an F1 score of 0.9852 in the training set, demonstrating superior performance in the testing set with an AUC of 0.9059 and F1 score of 0.9027. Key features identified through SHapley Additive exPlanations (SHAP) included chest tightness, aversion to cold, age, TCMSS, Short Form (36) Health Survey (SF-36), C-reactive protein (CRP), and lymphocyte ratio. The logistic regression-based nomogram demonstrated an AUC of 0.9479 and F1 score of 0.9384 in the testing set.

**Conclusion:**

This study utilized multicenter data and multiple ML algorithms to create a ML model for predicting TCM efficacy in Long COVID treatment. Furthermore, a logistic regression-based nomogram was developed to assist the model and improve decision-making efficiency in TCM applications for Long COVID management.

## 1 Introduction

Long COVID, also known as post-COVID syndrome, refers to a condition in which some individuals, after recovering from the acute phase of a COVID-19 infection, continue to experience persistent or recurrent symptoms that last longer than the typical recovery period. The World Health Organization (WHO) defines Long COVID as symptoms that persist for at least three months or longer following the acute phase of COVID-19 (1). The incidence of Long COVID is estimated to range from 10 to 30% in non-hospitalized cases and 50–70% in hospitalized cases. It is primarily characterized by symptoms such as fatigue, shortness of breath, dizziness, heart palpitations, insomnia, loss of smell, and poor appetite, affecting multiple systems including the respiratory, digestive, nervous, circulatory, and immune systems. These symptoms may be accompanied by various adverse outcomes and can persist for years or even a lifetime (2). According to a 2023 report by Centers for Disease Control and Prevention (CDC), 11% of American adults who previously contracted COVID-19 continue to experience Long COVID. The resulting limitations in daily activities affect quality of life and work capacity, potentially preventing working-age adults from maintaining employment (3). This places a substantial burden on individuals, healthcare systems, and the national economy.

The pathogenesis of Long COVID is not yet fully understood, but the predominant hypotheses include viral persistence and remnants in tissues, immune dysregulation, microbiome imbalances, and tissue damage resulting from chronic inflammation (4). There is currently no standardized treatment for Long COVID, with treatment primarily focusing on symptom relief and rehabilitation. These include antiviral drugs to clear the virus, anti-inflammatory drugs and immunosuppressants to alleviate systemic inflammation and suppress excessive immune responses, as well as rehabilitation therapies targeting specific symptoms, such as respiratory training, psychological support, cognitive and speech therapy, and physical rehabilitation. However, the effectiveness of current treatments remains uncertain, with a lack of mechanism-driven therapies targeting specific symptoms and standardized rehabilitation protocols. As a result, further research into diverse clinical approaches and models is needed to optimize treatment strategies, rehabilitation methods, and health services for Long COVID (5).

Traditional Chinese Medicine (TCM) has a history spanning thousands of years in the prevention and treatment of viral infectious pneumonia, with extensive clinical experience and effective methods (6). TCM follows a systemic and holistic approach, acting through multiple pathways on various targets (7), which aligns with the multisymptomatic and multisystemic characteristics of Long COVID. In the “Diagnosis and Treatment Program for Coronavirus Disease 2019 (COVID-19)” issued by the National Health Commission of the People's Republic of China (NHC), TCM is recommended for treatment during the recovery phase of COVID-19 (8). Research has shown that TCM can alleviate symptoms of Long COVID and significantly aid in the recovery of physiological functions (9). TCM provides a range of benefits, including neuroprotection, regulation of gastrointestinal and cardiopulmonary functions, and enhancement of immune function (10). However, in clinical practice, the applicability of TCM for treating Long COVID remains unclear, with no clear quantitative indicators. This lack of clarity makes it challenging for both physicians and patients to fully assess the effectiveness of TCM. Therefore, it is essential to collect large-scale, high-quality clinical data and apply precise analytical methods to identify the key characteristics of Long COVID patients who are suitable for TCM treatment. This will assist in clinical decision-making and support the broader application of TCM in treating Long COVID.

Machine Learning (ML) has shown significant advantages in predicting clinical outcomes, including diagnosis, treatment efficacy, and prognosis. Compared to traditional statistical methods, ML can manage complex variable interactions and non-linear relationships, leading to its growing use in clinical research, especially when the outcomes of interest are influenced by intricate associations among multiple factors (11). However, ML models are often viewed as “black boxes” due to their lack of transparency. Explainable Artificial Intelligence technologies enable clinicians and researchers to better understand the decision-making processes and outputs of ML algorithms, thereby supporting the wider adoption of ML in clinical practice (12). Therefore, leveraging ML methods to establish precise quantitative standards and determine the appropriate scope of TCM use can improve the accuracy of clinical decision-making, optimize patient treatment strategies, and facilitate the broader implementation of TCM practices (13, 14).

To our knowledge, previous studies developing clinical prediction models for TCM have often overlooked the inclusion of auxiliary examination indicators as potential factors in evaluating TCM efficacy (15, 16). While the clinical presentation and efficacy assessment of Long COVID predominantly rely on symptoms, certain auxiliary examination indicators may prove vital in predicting the effectiveness of TCM in treating Long COVID (17). This study leveraged case data from a clinical database, collecting baseline patient characteristics, clinical symptoms, scores, and auxiliary examination results. Five ML models were developed, including Support Vector Machine (SVM), Random Forest (RF), K-Nearest Neighbors (KNN), Extreme Gradient Boosting (XGBoost), and Neural Networks (NN). Through these models, we aim to identify the optimal predictive algorithm and utilize SHapley Additive exPlanations (SHAP) to interpret the models. The objective is to develop an explainable ML model, pinpoint key features associated with effective treatment, and assess the feasibility and clinical utility of the model in accurately predicting the efficacy of TCM for Long COVID.

Additionally, while ML models surpass traditional nomograms in predictive efficiency, the simplicity and interpretability of nomograms remain highly valuable for clinical decision-making and patient education (18). Thus, our secondary objective is to develop a high-performance nomogram using logistic regression, ensuring robust predictive accuracy. This nomogram would serve as a complement to the ML model, facilitating its broader adoption in clinical practice.

## 2 Methods

### 2.1 Data source and extraction

**Figure 1:**
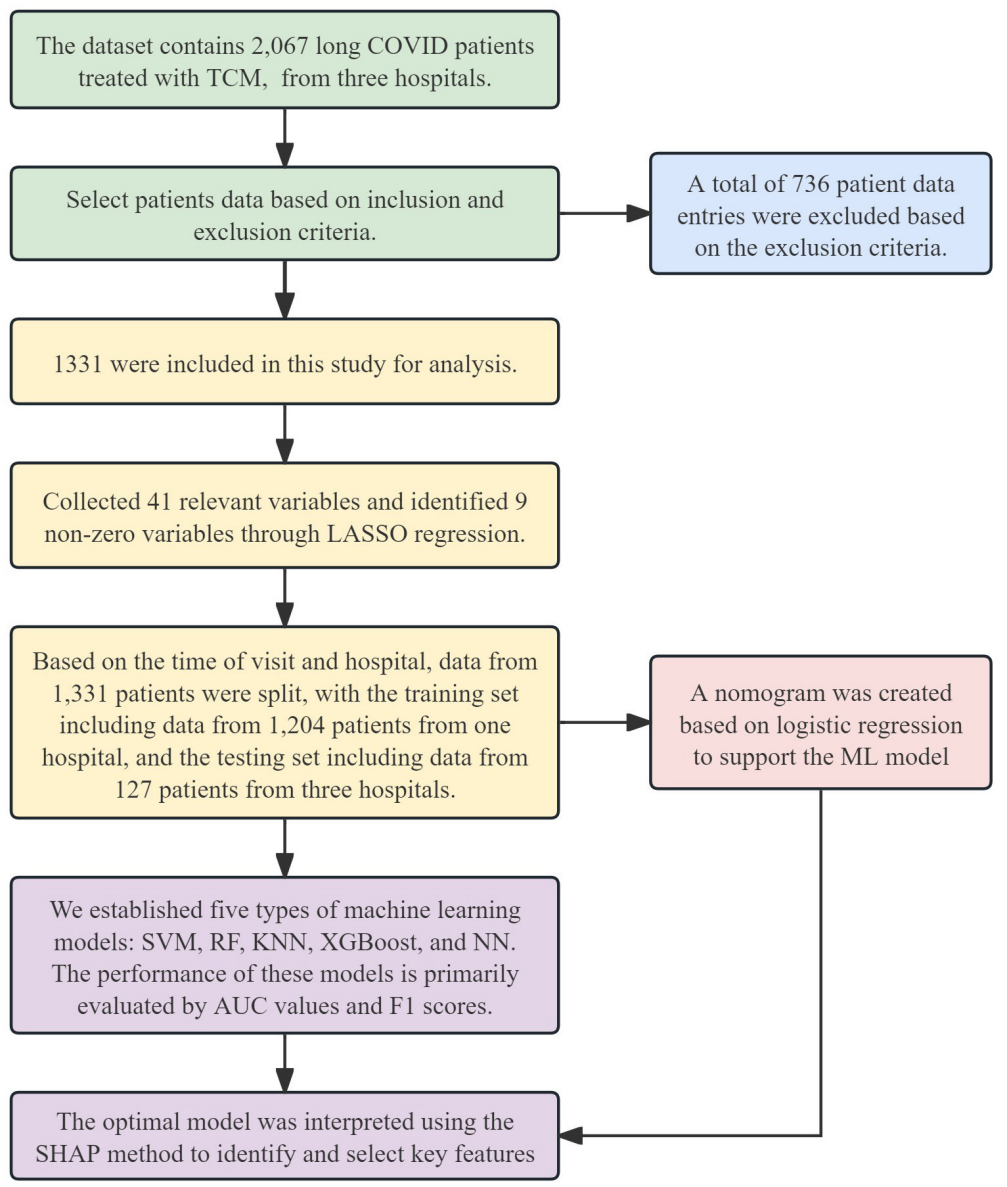
Flowchart of this study. TCM, Traditional Chinese Medicine; LASSO, Least Absolute Shrinkage and Selection Operator; SVM, Support Vector Machine; RF, Random Forest; KNN, K-Nearest Neighbors; XGBoost, Extreme Gradient Boosting; NN, Neural Network; AUC, Area Under the Curve; SHAP, SHapley Additive exPlanations; ML, machine learning.

The dataset for this study was sourced from the Long COVID databases of the Third Affiliated Hospital of Zhejiang Chinese Medical University (TAHZCMU), Jiaxing First People's Hospital (JFPH), and Haining People's Hospital (HPH). This study aims to extract data from the database on Long COVID cases treated with TCM for retrospective analysis, with the goal of developing and validating a predictive model for the efficacy of TCM. The study protocol was approved by the Ethics Committee of TAHZCMU (ZSLL-KY-2023-002-01). We followed the reporting guidelines in TRIPOD (transparent reporting of a multivariate prediction model for individual prognosis or diagnosis). The Flowchart of this study is outlined in Figure 1.

Data from 2,067 Long COVID patients treated with TCM were extracted from the database. These patients received treatment between December 2022 and February 2024 at TAHZCMU, JFPH, and HPH. All patients were from Zhejiang Province, China, and were treated using a sequential TCM therapy over a 14-day period. The detailed treatment protocol is shown in Table 1.

**Table 1:** Detailed Traditional Chinese Medicine treatment plan.

	**TCM prescription**	**Acupuncture points**	**Treatment methods**
Phase 1	**CRPRF:** Bupleurum (Chai Hu) 12 g, Scutellaria (Huang Qin) 9 g, Glehnia Root (Bei Sha Shen) 12 g, Pinellia Tuber (Jiang Ban Xia) 12 g, Fresh Ginger (Sheng Jiang) 6 g, Licorice (Gan Cao) 10 g, Fagopyrum Dibotrys (Jin Qiao Mai) 20 g, Scrophularia Root (Xuan Shen) 20 g, Platycodon Root (Jie Geng) 10 g, Belamcanda (She Gan) 12 g, Ophiopogon Root (Mai Dong) 12 g, Perilla Leaf (Zi Su Ye) 15 g, Aster Root (Zi Wan) 12 g, Coltsfoot Flower (Kuan Dong Hua) 12 g, and Reed Rhizome (Lu Gen) 30 g	DU14 (Dazhui), BL12 (Fengmen), RN22 (Tiantu), SJ5 (Waiguan), LU7 (Lieque), and ST40 (Fenglong)	The herbal medicine is decocted into 150 ml per packet, with one packet taken each morning and evening, for a total duration of 14 days. The acupuncture points are uniformly treated with Thumb-tack needle (ϕ0.25 × 1.3 mm), with an application duration of 24 h, administered 3 times per week for a total of 6 sessions
Phase 2	**YTRF:** Sun, Dried Ginseng (Sheng Shai Shen) 10 g, Ophiopogon Root (Mai Dong) 12 g, Schisandra (Wu Wei Zi) 5 g, Pinellia Tuber (Jiang Ban Xia) 9 g, Fresh Ginger (Sheng Jiang) 6 g, Honey, Fried Licorice (Zhi Gan Cao) 9 g, Lophatherum Stem (Dan Zhu Ye) 10 g, Rhodiola (Hong Jing Tian) 20 g, Dried Tangerine Peel (Chen Pi) 12 g, Stir, Fried Atractylodes (Chao Bai Zhu) 12 g, Agrimony (Xian He Cao) 30 g, Cinnamon Twig (Gui Zhi) 10 g, Perilla Leaf (Zi Su Ye) 12 g, Oroxylum Seed (Mu Hu Die) 6 g, and Mulberry Leaf (Sang Ye) 9 g	BL13 (Feishu), BL15 (Xinshu), RN4 (Guanyuan), KI3 (Taixi), PC6 (Neiguan), and HT7 (Shenmen)	

CRPRF, cough relieving and phlegm resolving formula; YTRF, Yin transformation and revitalization formula; DU, Du Meridian; BL, Badder Meridian; RN, Ren Meridian; SJ, Sanjiao Meridian; LU, Lung Meridian; ST, Stomach Meridian; KI, Kidney Meridian; PC, Pericardium Meridian; HT, Heart Meridian.

We included case data from patients who met the medical diagnostic criteria for Long COVID and received TCM treatment. The following patients were excluded: (1) those without follow-up records; (2) those with more than 20% missing clinical data in their follow-up records; (3) those with a treatment duration of < 14 days; (4) pregnant or breastfeeding women; (5) those participating in other clinical drug trials; and (6) those unable to complete the treatment or trial for any other reason.

Based on published research, clinical expertise, and practical considerations from factor evaluations in clinical practice, we identified potential factors from the case data related to the efficacy of TCM in treating Long COVID (19–21). For factors that were highly correlated, we either retained the most relevant one or combined them (22). The following factors were collected: First, 11 baseline characteristics, including age, gender, body mass index (BMI), hypertension, diabetes, hyperlipidemia, cerebrovascular disease, cardiovascular disease, smoking history, vaccination status, and secondary infection. Second, 18 clinical features and syndrome scores: cough, expectoration, nasal congestion, chest tightness, insomnia, sore throat, fatigue, aversion to cold, headache, myalgia, palpitation, anorexia, spontaneous/night sweats, fever, smell/taste problem, TCM syndrome score (TCMSS), Short Form (36) Health Survey (SF-36), and Pittsburgh Sleep Quality Index (PSQI). Third, 12 auxiliary examination indicators, comprising 3 pulmonary imaging markers: pulmonary nodule, pulmonary infection, and stable pulmonary lesion, as well as 9 blood and biochemical markers: C-reactive protein(CRP), white blood cells, platelets, neutrophils, lymphocyte ratio, red blood cell, hemoglobin, the ratio of Aspartate Aminotransferase to Alanine Aminotransferase (AST/ALT), and creatinine. Fourth, follow-up or feedback data after 14 days of TCM treatment.

As there are no standardized quantitative indicators for assessing the improvement of Long COVID, symptom relief remains the primary measure of treatment efficacy (23). In this study, treatment outcomes were evaluated by an experienced team of clinicians, following the World Health Organization (WHO) guidelines for Long COVID (24). Patients with improvement in two or more clinical symptoms, or with a TCMSS improvement of at least 2 points, were classified as having an effective treatment.

### 2.2 Model input features

A total of 41 variables were collected, and data processing was as follows: gender was coded as 1 for male and 0 for female; binary variables (clinical symptoms, medical history, pulmonary imaging features, and reinfection status) were coded as 1 for “yes” and 0 for “no.” The remaining variables were normalized using the min-max scaling method: *E*(*x*) = (*x* – min)/(max – min).

Variable selection was conducted using the least absolute shrinkage and selection operator (LASSO) method. The objective function of LASSO regression introduces a penalty term to the least squares method, shrinking some regression coefficients to zero, thereby enabling efficient feature selection. Potential risk factors were identified through LASSO regression, and the non-zero features were subsequently included in both the model and the nomogram. LASSO regression was implemented using the glmnet package in R (version 4.4.1; Foundation for Statistical Computing, Vienna, Austria).

### 2.3 Machine learning model and SHapley Additive exPlanations (SHAP)

The dataset was divided into training and testing sets based on the time of patient visits and the hospital of treatment. Five ML models were then developed for analysis: SVM model was implemented using the e1071 package in R, RF model with the randomForest package in R, KNN model with the kknn package in R, XGBoost model with the xgboost package in R, and NN model with the nnet package in R.

The evaluation of model performance included an analysis of Area Under the Curve (AUC), sensitivity, specificity, accuracy, and F1 score. Among these, AUC and F1 score were the primary metrics used for comparing model performance (25, 26). The most suitable ML model was then selected as the predictive model for this study.

We applied the SHAP method to interpret the ML models and evaluate the influence of different features on the prediction outcomes, identifying key features in the process. SHAP provides effective explanations of model performance at both the cohort and individual patient levels. By calculating and averaging the SHAP values for each feature across all patients, we were able to assess each feature's contribution to the model (27). The SHAP feature importance plot displays the global importance of these features, with higher mean absolute SHAP values indicating a greater contribution to model predictions. Additionally, the SHAP summary plot offers a clear visualization of each feature's specific impact on the predictions, with each point representing a feature value for an individual patient. We also generated SHAP dependence plots to further explain the influence of specific features on the prediction outcomes. The SHAP method was implemented using the shapviz package in R.

### 2.4 Nomogram

Perform logistic regression analysis on the training set to construct a corresponding nomogram, followed by validation and performance evaluation on the testing set. The logistic regression and nomogram are implemented using the rms package in R.

### 2.5 Statistical analyses

Continuous data were expressed as mean ± standard deviation or median with interquartile range (IQR), while categorical data were presented as frequency and percentage. For comparisons between groups, categorical variables were analyzed using the chi-square test, and continuous data were assessed using an independent samples *t*-test. All statistical analyses were conducted using IBM SPSS (Version 25.0; IBM, Armonk, NY, USA), with *P* < 0.05 (two-sided) considered statistically significant.

## 3 Results

### 3.1 Basic characteristics

This study included data from 1,331 Long COVID patients, with 1,155 classified as effective and 176 as ineffective. Although cases with more than 20% missing clinical data were excluded, some missing values still existed in the included patient records. The missing variables primarily involved Alanine Aminotransferase/Aspartate Aminotransferase (ALT/AST) and creatinine, each accounting for 3.5% of the total dataset. To address this, we used Random Forest regression to estimate the missing data. The mice package in R is used to impute missing values using the Random Forest regression algorithm, generating multiple imputed datasets. The first complete dataset is then selected from the imputed results and saved.

The completed dataset was divided based on the time and location of patient visits. Data from 1,204 patients treated at TAHZCMU between December 2022 and June 2023 were used as the training set, while data from 127 patients treated at TAHZCMU, JFPH, and HPH between June 2023 and February 2024 were used as the testing set. There were no significant differences in baseline characteristics between the training and test sets. A summary of patient characteristics in each group is provided in Table 2.

**Table 2:** Patient characteristics in the training and testing sets.

	**Training set (*****n*** = **1,204)**			**Testing set (*****n*** = **127)**		
**Characteristics**	**Ineffective (*n* = 157)**	**Effective (*n* = 1,047)**	***P*-value**	**Ineffective (*n* = 19)**	**Effective (*n* = 108)**	***P*-value**
Age (years)	46 (35, 62)	42 (34, 54)	0.029	47 (32, 52)	47.5 (34, 62.25)	0.4
BMI (kg/m^2^)	22.04 (20.57, 23.81)	21.88 (20.43, 23.91)	0.472	24.46 ± 4.12	23.65 ± 3.85	0.431
Sex (male = 1), *n* (%)			0.079			0.52
0	80 (51)	615 (59)		11 (58)	74 (69)	
1	77 (49)	432 (41)		8 (42)	34 (31)	
Hypertension, *n* (%)			0.855			0.623
0	149 (95)	986 (94)		17 (89)	101 (94)	
1	8 (5)	61 (6)		2 (11)	7 (6)	
Diabetes, *n* (%)			0.546			1
0	148 (94)	1,002 (96)		18 (95)	99 (92)	
1	9 (6)	45 (4)		1 (5)	9 (8)	
Hyperlipidemia, *n* (%)			0.429			0.623
0	156 (99)	1,044 (100)		17 (89)	101 (94)	
1	1 (1)	3 (0)		2 (11)	7 (6)	
Cerebrovascular disease, *n* (%)			0.184			0.481
0	150 (96)	1,021 (98)		18 (95)	105 (97)	
1	7 (4)	26 (2)		1 (5)	3 (3)	
Cardiovascular disease, *n* (%)			0.707			0.692
0	151 (96)	996 (95)		18 (95)	95 (88)	
1	6 (4)	51 (5)		1 (5)	13 (12)	
Smoking history, *n* (%)			0.916			0.13
0	150 (96)	1,006 (96)		12 (63)	87 (81)	
1	7 (4)	41 (4)		7 (37)	21 (19)	
Vaccination status, *n* (%)			0.885			0.779
0	14 (9)	101 (10)		4 (21)	29 (27)	
1	143 (91)	946 (90)		15 (79)	79 (73)	
Secondary infection, *n* (%)			0.558			0.313
0	98 (62)	624 (60)		5 (26)	45 (42)	
1	59 (38)	423 (40)		14 (74)	63 (58)	
Cough, *n* (%)			0.004			0.017
0	114 (73)	630 (60)		12 (63)	34 (31)	
1	43 (27)	417 (40)		7 (37)	74 (69)	
Expectoration, *n* (%)			< 0.001			0.044
0	139 (89)	756 (72)		15 (79)	55 (51)	
1	18 (11)	291 (28)		4 (21)	53 (49)	
Nasal congestion, *n* (%)			< 0.001			0.31
0	125 (80)	680 (65)		15 (79)	69 (64)	
1	32 (20)	367 (35)		4 (21)	39 (36)	
Chest tightness, *n* (%)			< 0.001			0.337
0	139 (89)	744 (71)		14 (74)	90 (83)	
1	18 (11)	303 (29)		5 (26)	18 (17)	
Insomnia, *n* (%)			0.557			0.518
0	128 (82)	877 (84)		15 (79)	91 (84)	
1	29 (18)	170 (16)		4 (21)	17 (16)	
Sore throat, *n* (%)			0.401			0.699
0	130 (83)	833 (80)		11 (58)	54 (50)	
1	27 (17)	214 (20)		8 (42)	54 (50)	
Fatigue, *n* (%)			0.749			1
0	101 (64)	691 (66)		12 (63)	71 (66)	
1	56 (36)	356 (34)		7 (37)	37 (34)	
Aversion to cold, *n* (%)			< 0.001			0.306
0	151 (96)	883 (84)		18 (95)	91 (84)	
1	6 (4)	164 (16)		1 (5)	17 (16)	
Headache, *n* (%)			0.042			0.361
0	152 (97)	962 (92)		17 (89)	83 (77)	
1	5 (3)	85 (8)		2 (11)	25 (23)	
Myalgia, *n* (%)			0.701			0.081
0	141 (90)	954 (91)		15 (79)	100 (93)	
1	16 (10)	93 (9)		4 (21)	8 (7)	
Palpitation, *n* (%)			0.597			0.762
0	148 (94)	971 (93)		16 (84)	84 (78)	
1	9 (6)	76 (7)		3 (16)	24 (22)	
Anorexia, *n* (%)			0.082			0.213
0	141 (90)	983 (94)		16 (84)	100 (93)	
1	16 (10)	64 (6)		3 (16)	8 (7)	
Spontaneous/night sweats, *n* (%)			0.019			0.692
0	154 (98)	971 (93)		18 (95)	95 (88)	
1	3 (2)	76 (7)		1 (5)	13 (12)	
Fever, *n* (%)			0.556			0.523
0	152 (97)	999 (95)		17 (89)	87 (81)	
1	5 (3)	48 (5)		2 (11)	21 (19)	
Smell/taste problem, *n* (%)			0.051			1
0	155 (99)	993 (95)		19 (100)	103 (95)	
1	2 (1)	54 (5)		0 (0)	5 (5)	
TCM syndrome score (points)	4 (2, 5)	7 (3, 10)	< 0.001	3 (3, 4)	6 (5, 8)	< 0.001
SF-36 (points)	124 (119, 132)	96 (79.95, 111)	< 0.001	128 (126, 137)	99.08 (69, 119.29)	< 0.001
PSQI (points)	8 (4, 10)	7 (2, 12)	0.698	4 (2, 6.5)	7 (4, 10.25)	0.011
Pulmonary nodule, *n* (%)			0.002			0.517
0	110 (70)	595 (57)		13 (68)	62 (57)	
1	47 (30)	452 (43)		6 (32)	46 (43)	
Pulmonary infection, *n* (%)			0.405			1
0	147 (94)	1,000 (96)		19 (100)	103 (95)	
1	10 (6)	47 (4)		0 (0)	5 (5)	
Stable pulmonary lesion, *n* (%)			0.469			0.59
0	148 (94)	1,004 (96)		19 (100)	102 (94)	
1	9 (6)	43 (4)		0 (0)	6 (6)	
C-reactive protein (mg/L)	5.89 (3.48, 8.8)	17.34 (9.11, 24.99)	< 0.001	3.61 (2.32, 7.16)	13.43 (6.97, 24.72)	< 0.001
White blood cells (10^9^/L)	7.94 (5.71, 10.43)	7.81 (5.63, 10.07)	0.484	7.93 (7.2, 8.68)	6.99 (5, 10.12)	0.216
Platelets (10^9^/L)	248 (182, 320)	250 (182, 318.5)	0.876	267 (187, 329)	234 (180.75, 303.5)	0.339
Neutrophils (10^9^/L)	5.37 (2.87, 7.52)	5.9 (3.96, 7.84)	0.013	6.34 (4.84, 7.76)	5.9 (3.8, 8.11)	0.521
Lymphocyte ratio (%)	31.9 (27.2, 36.2)	27.55 (21.88, 33.72)	< 0.001	32.36 ± 6.8	23.27 ± 7.39	< 0.001
Red blood cell (10^12^/L)	4.6 (3.9, 5.3)	4.6 (3.8, 5.3)	0.428	4.4 (4.04, 5.56)	4.5 (3.98, 4.95)	0.605
Hemoglobin (g/L)	130 (112, 146)	130 (114, 146)	0.899	122 (110.5, 145)	130.5 (118.75, 149)	0.309
AST/ALT	1.05 (0.89, 1.26)	1.08 (0.88, 1.28)	0.782	1.06 (0.86, 1.25)	1.15 (0.92, 1.32)	0.433
Creatinine (μmol/L)	82.9 (61.1, 104.4)	78.9 (59.55, 99.4)	0.118	86 (68, 106)	82 (62, 97.5)	0.374

Data are shown as mean ± standard deviation (normal data) or median (Q1, Q3) (non-normal data) or n (%) (classify data). BMI, body mass index; TCM, Traditional Chinese Medicine; SF-36, Short Form (36) Health Survey; PSQI, Pittsburgh Sleep Quality Index; AST, Aspartate Aminotransferase; ALT, Alanine Aminotransferase.

### 3.2 Lasso regression results

**Figure 2:**
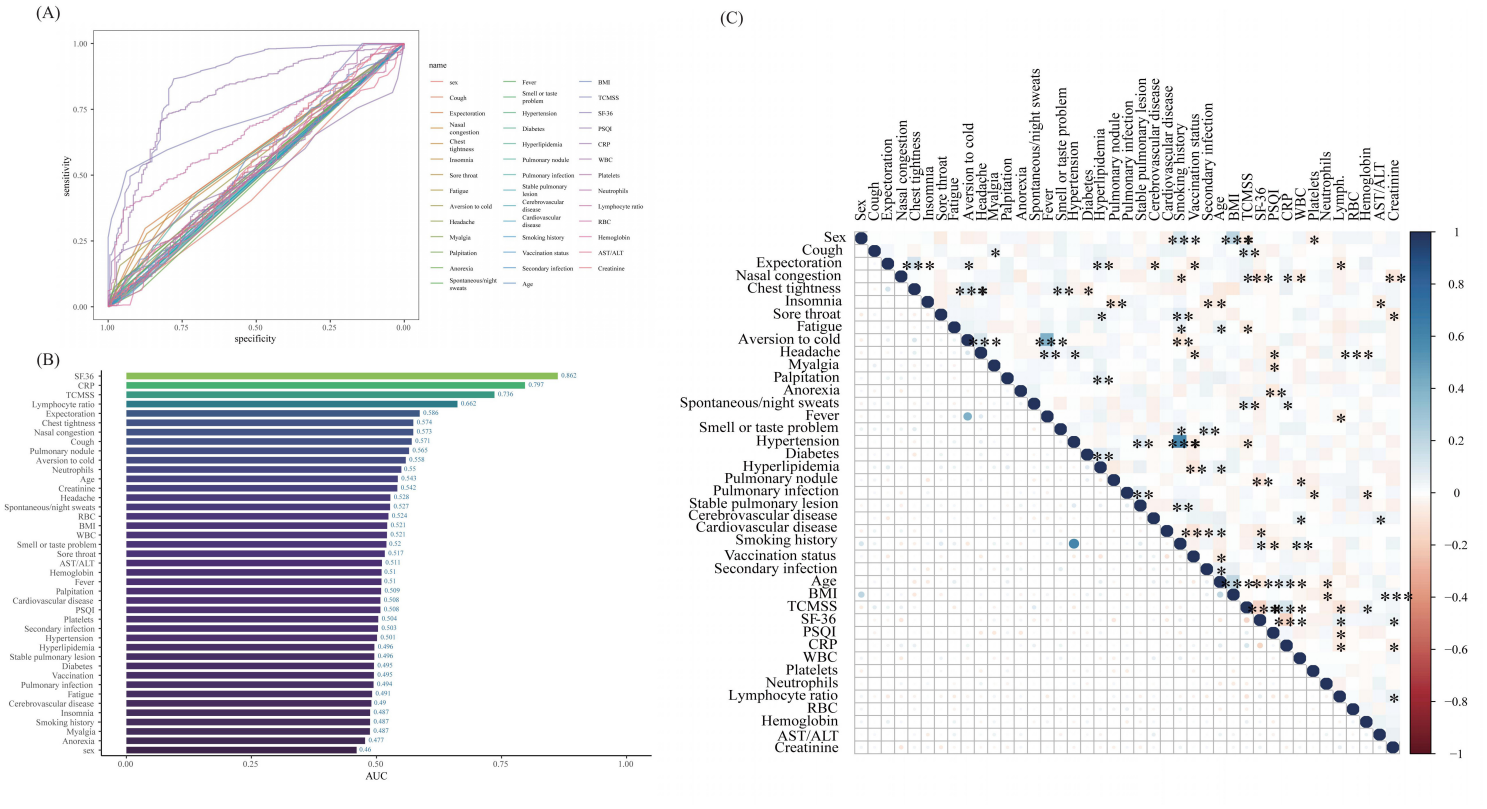
The results of the ROC curve, AUC value, and correlation analysis. **(A)** Receiver Operating Characteristic (ROC) curve plot; **(B)** Area Under the Curve (AUC) bar chart; **(C)** Correlation matrix heatmap. BMI, body mass index; TCMSS, Traditional Chinese Medicine syndrome score; SF-36, Short Form (36) Health Survey; PSQI, Pittsburgh Sleep Quality Index; CRP, C-reactive protein; WBC, White blood cells; RBC, Red blood cells; AST/ALT, the ratio of Aspartate Aminotransferase to Alanine Aminotransferase. *p < 0.05, **p < 0.01, ***p < 0.001.

Prior to conducting Lasso regression, we first performed Receiver Operating Characteristic (ROC) curve analysis for individual features (Figure 2A) and generated a bar chart representing the AUC values (Figure 2B). The results indicated that several features had relatively high AUC values: SF-36 (AUC = 0.862), CRP (AUC = 0.797), TCMSS (AUC = 0.736), lymphocyte ratio (AUC = 0.662), expectoration (AUC = 0.586), chest tightness (AUC = 0.574), nasal congestion (AUC = 0.573), cough (AUC = 0.571), pulmonary nodule (AUC = 0.571), aversion to cold (AUC = 0.558), neutrophils (AUC = 0.550), age (AUC = 0.543), and creatinine (AUC = 0.542). These features are likely candidates for inclusion in the model. To further refine the model, we conducted a correlation analysis of all features (Figure 2C) to eliminate highly correlated factors that might negatively impact model performance.

**Figure 3:**
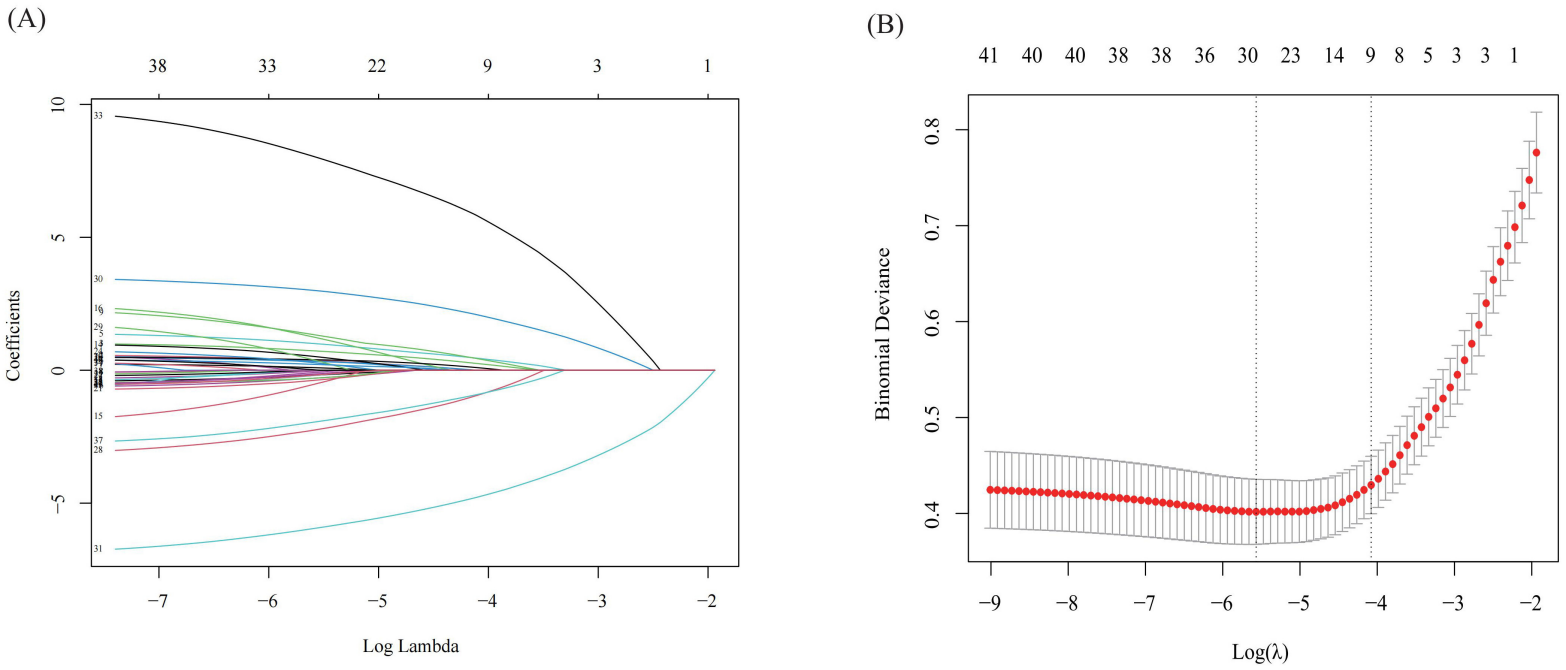
The results of Lasso regression. **(A)** LASSO Coefficient Path Plot; **(B)** Cross-Validation Error Plot for LASSO.

The optimal parameter (lambda) for LASSO regression was determined using 10-fold cross-validation, with the best value indicated by the minimum criteria and the 1-SE (standard error) of the minimum criteria, represented by dashed lines (Figures 3A, B). Our LASSO regression results identified nine significant variables: SF-36, CRP, TCMSS, lymphocyte ratio, expectoration, chest tightness, Pulmonary nodule, aversion to cold, and age. Due to the low correlation among these variables, they were all included in the final model.

### 3.3 Model evaluation and interpretation

**Figure 4:**
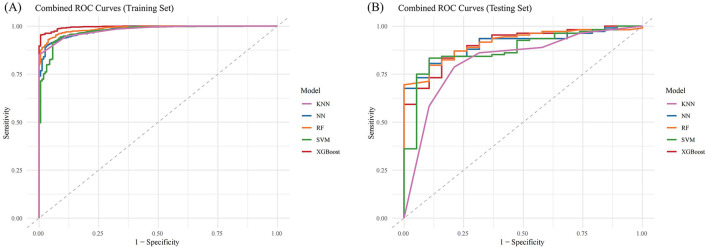
Combined ROC Curves for Multiple Machine Learning Models. **(A)** ROC curves for training sets; **(B)** ROC curves for testing sets. KNN, K-Nearest Neighbors; NN, Neural Network; RF, Random Forest; SVM, Support Vector Machine; XGBost, Extreme Gradient Boosting.

When evaluating the performance of the ML models on the training set, XGBoost and RF showed the highest AUC values (XGBoost: 0.9957, 95% CI: 0.9934–0.9981; RF: 0.9871, 95% CI: 0.9819–0.9924) (Figure 4A and Table 3). The F1 score was used to further compare model performance, as it is well-suited for imbalanced datasets, reflecting a balance between precision (positive predictive value) and recall (sensitivity) (25, 26). The XGBoost model achieved the highest F1 score (0.9852, 95% CI: 0.9777–0.9921). In the testing set, XGBoost continued to perform well, with an AUC of 0.9059 (95% CI: 0.8437–0.9682) and the highest F1 score of 0.9027 (95% CI: 0.8258–0.9485). In contrast, the RF model underperformed, with a lower F1 score (0.8197, 95% CI: 0.7315–0.8749), suggesting weaker generalization ability and potential overfitting (Figure 4B and Table 4) (28). As a result, the XGBoost model was selected for downstream analysis.

**Table 3:** Evaluation of model performance in the training set.

	**AUC (95% CI)**	**Sensitivity (95% CI)**	**Specificity (95% CI)**	**Accuracy (95% CI)**	**F1 score (95% CI)**
XGBoost	0.9957 (0.9934, 0.9981)	0.9952 (0.9882, 0.9982)	0.8535 (0.7862, 0.9030)	0.9767 (0.9666, 0.9845)	0.9852 (0.9777, 0.9921)
NN	0.9797 (0.9724, 0.9871)	0.8997 (0.8795, 0.9169)	0.9682 (0.9234, 0.9882)	0.9086 (0.8909, 0.9243)	0.9448 (0.9302, 0.9561)
RF	0.9871 (0.9819, 0.9924)	0.9303 (0.9127, 0.9446)	0.9618 (0.9150, 0.9844)	0.9344 (0.9189, 0.9477)	0.9610 (0.9470, 0.9705)
SVM	0.9737 (0.9629, 0.9845)	0.9207 (0.9023, 0.9360)	0.9363 (0.8828, 0.9673)	0.9228 (0.9062, 0.9372)	0.9540 (0.9403, 0.9651)
KNN	0.9794 (0.9729, 0.9859)	0.8500 (0.8266, 0.8708)	1.0000 (0.9702,1.0000)	0.8696 (0.8493, 0.8881)	0.9189 (0.9046, 0.9308)

Data are shown as n (95%CI); sensitivity = TP/(TP + FN); specificity = TN/(TN + FP); accuracy = (TP + TN)/(TP + TN + FP + FN); F1 = (2 ^*^ TP)/(2 ^*^ TP + FP + FN). FN, false negatives; FP, false positives; TN, true negatives; TP, true positives.

**Table 4:** Evaluation of the model performances in the testing set.

	**AUC (95% CI)**	**Sensitivity (95% CI)**	**Specificity (95% CI)**	**Accuracy (95% CI)**	**F1 score (95% CI)**
XGBoost	0.9059 (0.8437, 0.9682)	0.8611 (0.7781, 0.9176)	0.7368 (0.4858, 0.8988)	0.8425 (0.7673, 0.9011)	0.9027 (0.8258, 0.9485)
NN	0.905 (0.8492, 0.9607)	0.7222 (0.6264, 0.8020)	0.9474 (0.7189, 0.9972)	0.7559 (0.6718, 0.8277)	0.8342 (0.7464, 0.8896)
RF	0.9125 (0.8582, 0.9669)	0.6944 (0.5973, 0.7775)	1.0000 (0.7908,1.0000)	0.7402 (0.6549, 0.8139)	0.8197 (0.7315, 0.8749)
SVM	0.8782 (0.8021, 0.9543)	0.6759 (0.4918, 0.6685)	0.9474 (0.7189, 0.9972)	0.7165 (0.6298, 0.7929)	0.8022 (0.7095, 0.8645)
KNN	0.8243 (0.7225, 0.9261)	0.5833 (0.4845, 0.6762)	0.8947 (0.6546, 0.9816)	0.6299 (0.5398, 0.7139)	0.7283 (0.6254, 0.8054)

Data are shown as n (95%CI); sensitivity = TP/(TP + FN); specificity = TN/(TN + FP); accuracy = (TP + TN)/(TP + TN + FP + FN); F1 = (2 ^*^ TP)/(2 ^*^ TP + FP + FN). FN, false negatives; FP, false positives; TN, true negatives; TP, true positives.

**Figure 5:**
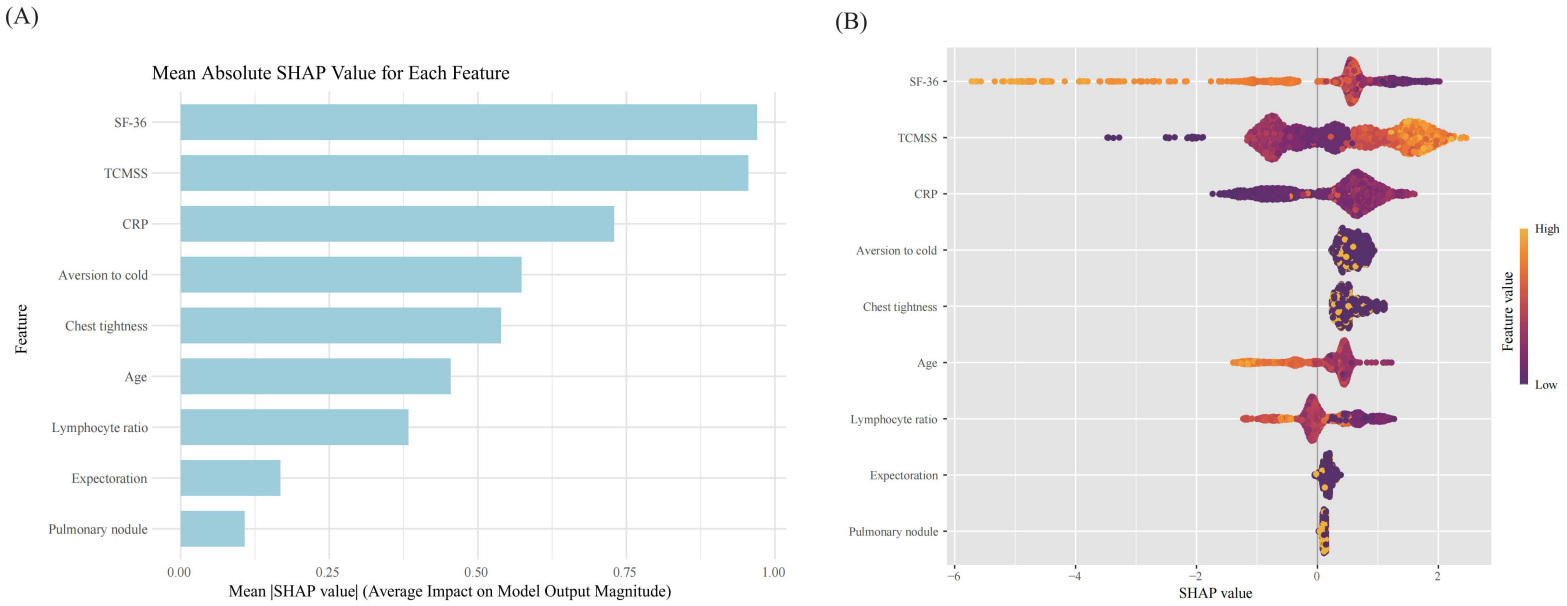
SHAP value of each feature in the model. **(A)** SHAP feature importance shown according to the mean absolute SHAP value of each feature; **(B)** SHAP summary plot showing the distribution of the SHAP values of each feature. SF-36, Short Form (36) Health Survey; TCMSS, Traditional Chinese Medicine syndrome score; CRP, C-reactive protein.

The SHAP feature importance for the XGBoost model is shown in Figure 5A, with features ranked by mean absolute SHAP values from highest to lowest. The key features identified were SF-36, TCMSS, CRP, aversion to cold, chest tightness, age, and lymphocyte ratio. The SHAP summary plot (Figure 5B) illustrates the influence of each feature on the model's predictions. A higher SHAP value indicates a greater likelihood of TCM being effective in treating Long COVID. For instance, patients with a lower lymphocyte ratio responded better to TCM treatment compared to those with a higher ratio. Similarly, patients with symptoms like chest tightness and aversion to cold showed a better response to TCM treatment than those without these symptoms.

**Figure 6:**
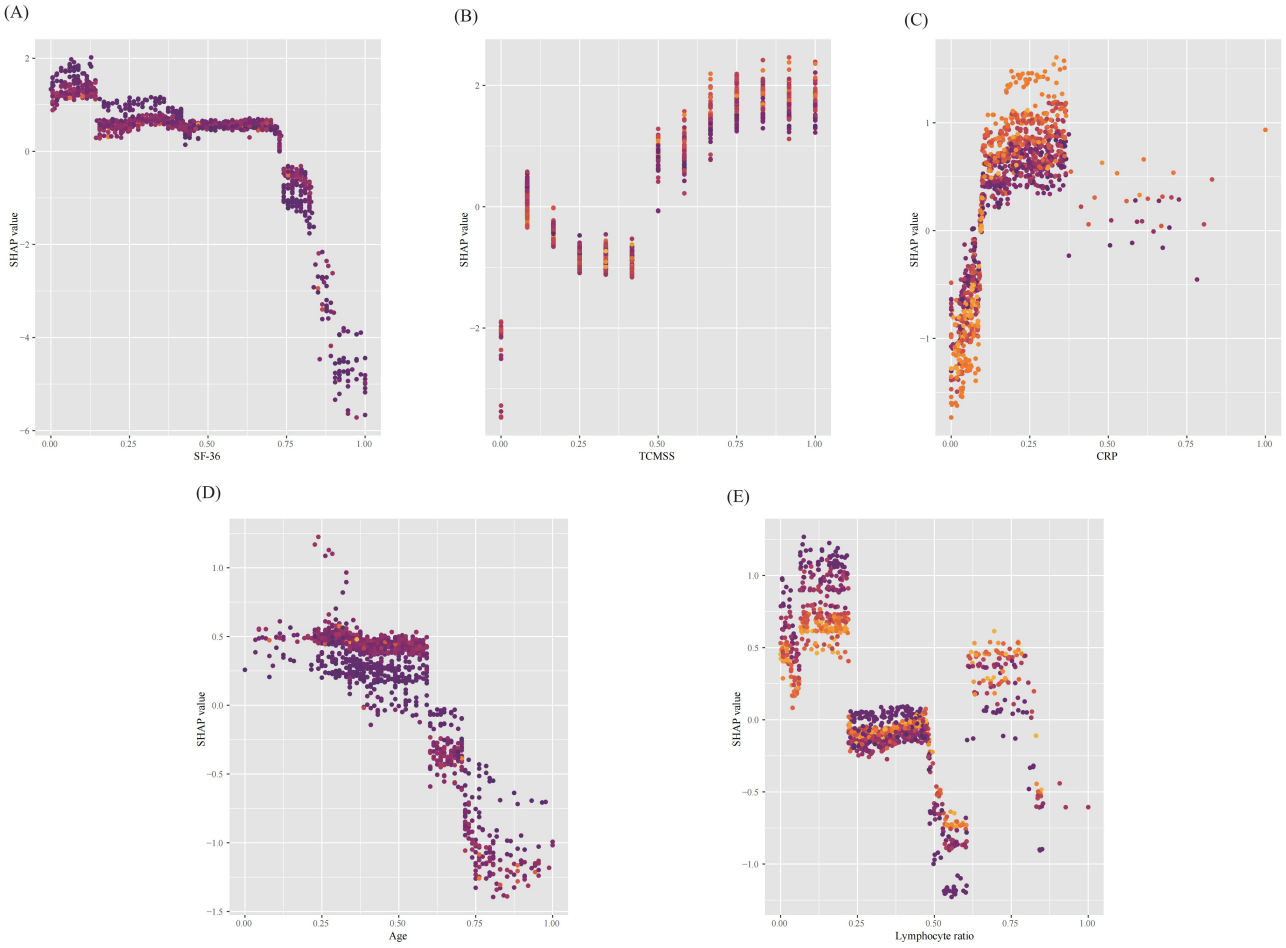
SHAP dependence plots of continuous features in the model. **(A)** Short Form (36) Health Survey (SF-36), **(B)** Traditional Chinese Medicine (TCM) syndrome score, **(C)** C-reactive protein (CRP), **(D)** age and **(E)** Lymphocyte ratio. The *y*-axis represents the SHAP values of features, and the values of certain features are shown in the *x*-axis, continuous variables were standardized using the min–max scaling method, resulting in values between 0 and 1. Each dot represents a SHAP value for a feature per patient, and color from light to dark represents the feature's value from high to low. SHAP values for specific features exceeding zero represent an increased probability of Traditional Chinese Medicine being effective in treating long COVID. SF-36, Short Form (36) Health Survey; TCMSS, Traditional Chinese Medicine syndrome score; CRP, C-reactive protein.

Additionally, the SHAP dependence plot (Figure 6) demonstrates the impact of individual continuous variables on the prediction of TCM treatment efficacy. Lower values for age, SF-36, and lymphocyte ratio (decreasing *x*-axis values) are associated with higher SHAP values, while increases in TCM syndrome score and CRP (increasing *x*-axis values) are also linked to higher SHAP values, indicating a greater likelihood of improvement following TCM treatment (increasing *y*-axis values).

### 3.4 Interpretation of ML models at the patient level

When evaluating the contribution of each feature to individual patients using the XGBoost model, we applied the SHAP method to randomly interpret the prediction results for two individual patients. The color represents each feature's contribution, with red indicating a positive contribution and blue indicating a negative contribution. The length of the color bar reflects the strength of the contribution. *E*[*f* (*x*)] is the baseline prediction value in the SHAP method, representing the average prediction of the model, typically the mean of the predictions across all samples. It serves as the reference or baseline against which the contribution of each feature to the model's output is measured. The formula is given by *E*[*f* (*x*)]$=1/N\;\overset{N}{\underset{i=1}{\sum }}fx_{i}$, where *N* is the number of samples in the dataset, and *fx*_*i*_ is the prediction value for the *i*-th sample (12). If the final predicted value *f* (*x*) exceeds the baseline prediction value *E*[*f* (*x*)], the case is classified as belonging to the effective group; if *f* (*x*) is lower than *E*[*f* (*x*)], the case is classified as belonging to the ineffective group.

**Figure 7:**
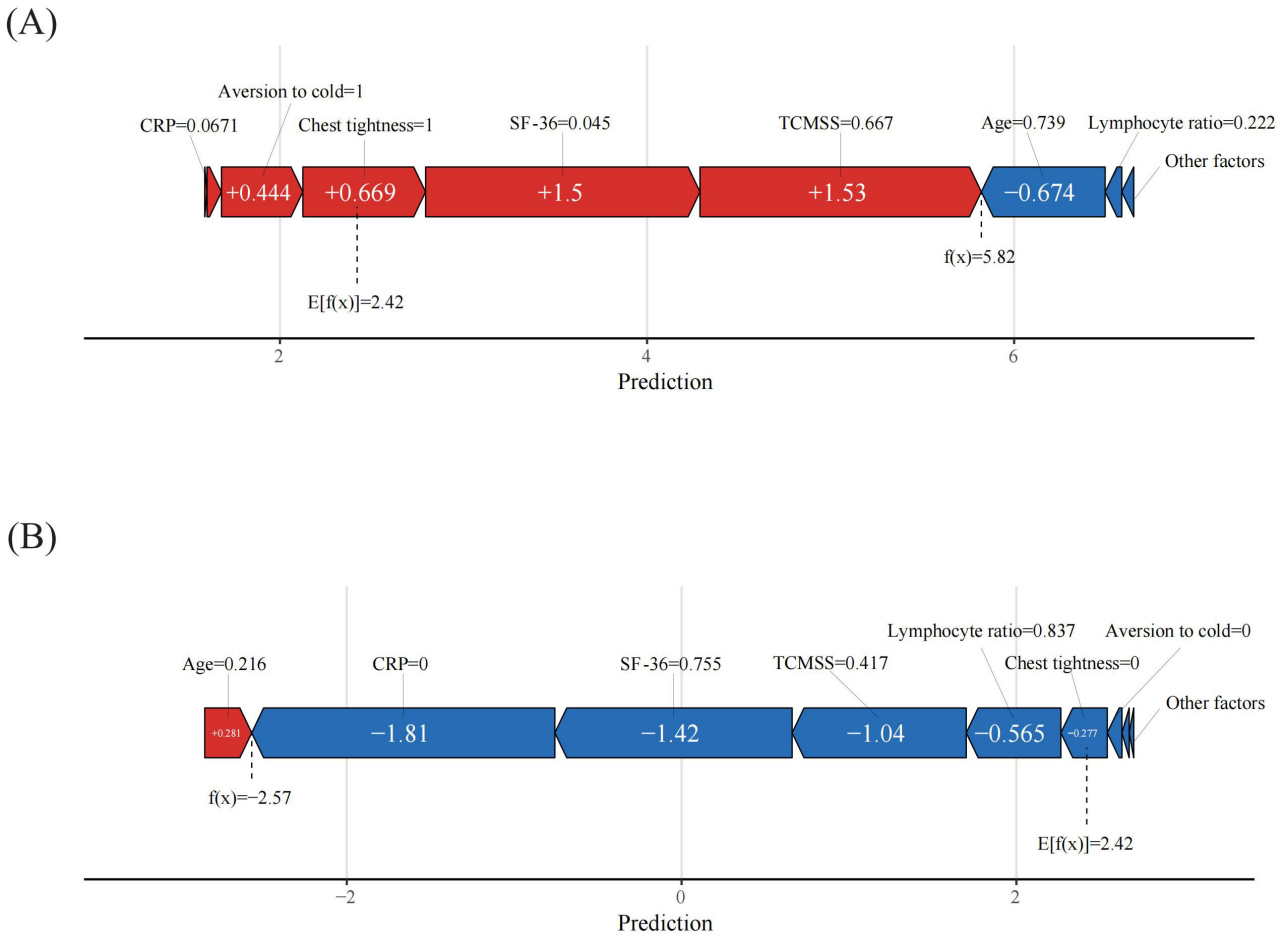
Patient-level SHAP force plots. **(A)** True positive patient, **(B)** True negative patient. The color represents the contributions of each feature, with red being positive and blue being negative. The length of the color bar represents the contribution strength.

As shown in Figure 7A, the predicted value for the current case, *f* (*x*) = 5.82, is much higher than the baseline prediction *E*[*f* (*x*)] = 2.42. In this case, factors such as CRP = 0.067 (indicating inflammation), aversion to cold = 1 (yes), chest tightness = 1 (yes), SF-36 = 0.045 (indicating poor quality of life), and TCM syndrome score = 0.667 (indicating poor health) all positively contributed to the prediction of TCM treatment effectiveness.

Similarly, in Figure 7B, the case was classified as ineffective, with a predicted value *f* (*x*) = −2.57, far lower than the baseline prediction value. In this case, CRP = 0 (no inflammation), SF-36 = 0.755 (indicating higher quality of life), TCM syndrome score = 0.417 (indicating better health), lymphocyte ratio = 0.837 (slightly above normal), chest tightness = 0 (no), and aversion to cold = 0 (no), all contributed to the prediction of treatment ineffectiveness.

### 3.5 Nomogram

**Figure 8:**
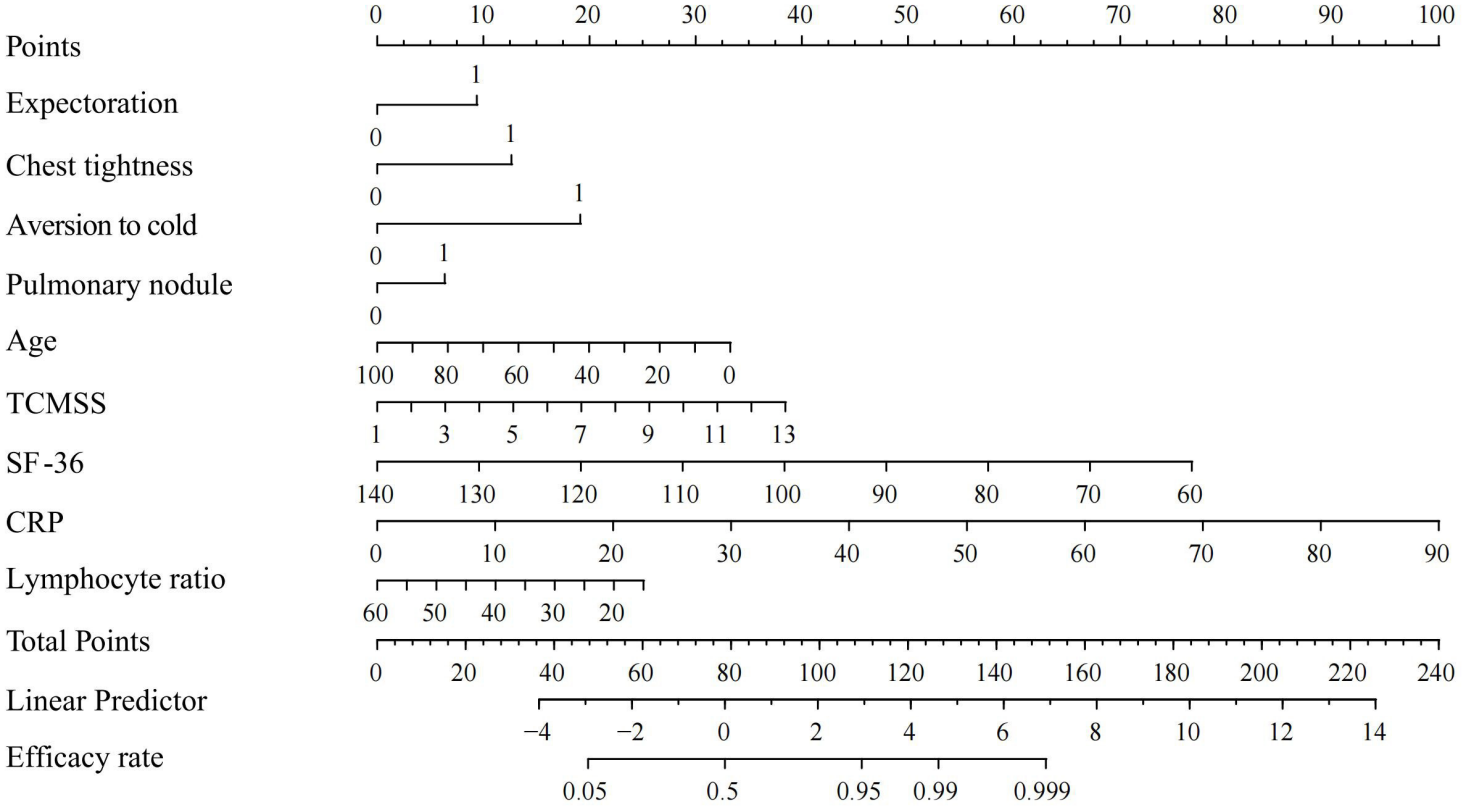
Nomogram for logistic regression. SF-36, Short Form (36) Health Survey; TCMSS, Traditional Chinese Medicine syndrome score; CRP, C-reactive protein.

In the training set, the nomogram developed from the logistic regression analysis (Figure 8) achieved an AUC of 0.9436 (95% CI, 0.9242–0.9629), an accuracy of 0.9228 (95% CI, 0.9058–0.9369), and an F1 score of 0.9562 (95% CI, 0.9470–0.9649). The nomogram was then evaluated on the testing set, where it achieved an AUC of 0.9479 (95% CI: 0.9092–0.9865), an accuracy of 0.8976 (95% CI: 0.8281–0.9422), and an F1 score of 0.9384 (95% CI: 0.9005–0.9694), demonstrating strong predictive performance.

## 4 Discussion

Long COVID predominantly manifests through a series of debilitating symptoms, encompassing multi-system dysfunction (23). Several studies have demonstrated that TCM can alleviate symptoms such as dyspnea, reduced respiratory rate, fatigue, and myalgia associated with Long COVID, while enhancing overall quality of life. Moreover, TCM has been shown to improve infection markers, reduce lung fibrosis, enhance coagulation function and myocardial recovery, strengthen immune function, and inhibit viral replication (9, 29). In the early recovery stage of Long COVID-19, TCM focuses on alleviating fatigue, improving neurological symptoms, and regulating the spleen and stomach function. In the chronic persistent stage, TCM emphasizes comprehensive intervention for multi-system symptoms, with long-term treatment aimed at restoring the body's functional balance (6, 10). The TCM treatment plan for Long COVID used in this study was developed based on the most commonly prescribed formulas in the Zhejiang Provincial Medical Information System (HALO System: Copyright 1999–2024 Lianzhong Zhihui) and tailored to the pathogenesis of Long COVID patients in Zhejiang, characterized by “Yang Qi deficiency with Qi and Yin depletion.” This plan was formed through consensus among TCM experts from multiple hospitals. A sequential treatment approach was adopted to enhance efficacy and reduce side effects (30). Preliminary cohort studies and multicenter randomized controlled trials have demonstrated that, compared to conventional therapies (e.g., corticosteroids or metoprolol), this treatment plan offers superior improvement in clinical symptoms, with comparable effects on inflammatory markers and pulmonary imaging outcomes [unpublished observation].

To better promote the application of TCM in Long COVID treatment, we applied various ML models for data analysis and found that the XGBoost model performed best. This led to the development of the first ML model to predict the efficacy of TCM in treating Long COVID. XGBoost is a highly efficient ML algorithm built on a gradient boosting framework, specifically designed to handle sparse data. The core advantages of XGBoost are as follows: First, it integrates the gradient boosting framework with deep optimization of computational systems, overcoming the computational efficiency bottleneck while maintaining the interpretability of tree-based models. Second, through second-order derivative optimization and multiple regularization designs, it achieves a better balance in the bias-variance trade-off. Additionally, it incorporates dedicated mechanisms to handle specific data characteristics (sparsity, missing values, and high dimensionality). These innovations enable XGBoost to demonstrate more robust predictive performance compared to other models, especially in business scenarios where feature interactions are complex but data volumes are limited (31). Among various ML models evaluated, XGBoost consistently outperformed others in both the training and test sets, exhibiting superior generalization ability. This indicates that XGBoost effectively captures the intricate features of Long COVID patients, enabling accurate predictions of TCM treatment outcomes. In the feature selection of this model, although the AUC values for the pulmonary nodule and aversion to cold features are relatively low, they remain above 0.5, indicating that these features have some predictive power. In the LASSO regression, features with significant impact are selected through the application of L1 regularization, and these features are chosen as non-zero variables, which are still considered valuable for model optimization.

Although logistic regression underperformed compared to the XGBoost model, the complexity of ML algorithms may impede understanding between healthcare providers and patients (32). This led us to develop a nomogram using logistic regression as a complementary tool to the ML model, making it more practical for clinicians and helping patients better understand the necessity of treatment. Although nomograms have certain limitations in handling complex non-linear relationships and large datasets compared to ML models like XGBoost (18), the nomogram demonstrated a high AUC and F1 score in the test set, indicating strong accuracy in predicting positive outcomes for Long COVID patients treated with TCM. Overall, the nomogram evaluation suggests that patients with total points exceeding 152, or a linear predictor value above 7, are more likely to experience improvement with TCM treatment.

In this study, we used LASSO regression to select nine risk factors for inclusion in the model. By further calculating the SHAP values for each feature in the model, we evaluated the contribution of different features to the model's predictions and identified seven key factors that play a critical role in predicting the efficacy of TCM for Long COVID treatment. These features include SF-36, TCMSS, CRP, aversion to cold, chest tightness, age, and lymphocyte ratio, all of which are easily assessable in clinical practice.

The SF-36 is a widely used standardized questionnaire that assesses eight dimensions, including physical functioning, social functioning, and mental health. It effectively evaluates the quality of life and health status in COVID and Long COVID patients (33). The TCMSS is a quantitative tool used to assess TCM syndromes, helping to objectify and standardize subjective diagnostic indicators. It serves as a key measure for evaluating the efficacy of TCM and summarizes the patient's physical condition at a particular stage of illness (34). Together, SF-36 and the TCM syndrome score provide valuable insights into the effectiveness of TCM in improving overall health and quality of life.

The symptom of aversion to cold is often linked to neurological dysfunction or vascular abnormalities (35), while chest tightness is commonly associated with cardiopulmonary dysfunction, particularly issues related to cardiac or vascular abnormalities and lung fibrosis (36, 37). This suggests that TCM may play a significant role in improving the neurological, circulatory, and respiratory functions affected by Long COVID. Furthermore, the correlation between increased age and reduced treatment efficacy may be attributed to immunosenescence, characterized by diminished anti-inflammatory mechanisms and persistent chronic inflammation, leading to slower recovery post-infection (38).

Additionally, this study identified two auxiliary diagnostic indicators that contribute to efficacy prediction, which have been largely overlooked in previously published TCM-related prediction models. CRP is a key marker of inflammation, sensitively reflecting the inflammatory state of Long COVID patients. Elevated CRP levels are typically associated with acute and chronic inflammatory processes (39). The lymphocyte ratio, reflecting immune function, represents a critical immune cell population involved in fighting infections and clearing pathogens. It plays an essential role during both the pathological process and recovery stages of viral diseases. A lower lymphocyte ratio often indicates impaired immune function (40, 41). While the mechanisms underlying these improvements require further rigorous biological research, their predictive value in treatment efficacy is already evident.

Therefore, this study demonstrates that despite the complexity of Long COVID's pathogenesis and the uncertainty surrounding treatment strategies, key features identified through ML models can reflect these underlying pathological processes. This provides a theoretical basis and predictive value for the application of TCM in treating Long COVID patients. Furthermore, the identification of these features may guide future efforts to explore and optimize TCM treatment regimens, addressing the diverse pathological changes associated with Long COVID.

The ML model developed in this study provides predictive information based on patients' clinical data, aimed at assisting healthcare providers in identifying Long COVID patients suitable for TCM treatment. When the model's predicted value exceeds the baseline prediction and the Nomogram's efficacy accuracy is >0.999, it suggests a higher likelihood of the patient benefiting from TCM treatment for Long COVID. However, the final validation of treatment effectiveness still requires further clinical research. Additionally, a dynamic calibration mechanism should be established in clinical practice to continuously monitor the model's predictive performance, ensuring its consistency with actual treatment outcomes. At the same time, healthcare providers should have a certain understanding of the principles behind machine learning models, as this not only helps doctors make more scientifically informed diagnostic decisions but also enables patients to better understand the necessity of the treatment plan.

However, this study has various limitations. First, although our ML model was trained on a large-scale database from a tertiary medical center and tested using multiple external databases, all case data were derived from Zhejiang Province, China, necessitating caution when applying these results to other regions; Second, as the study was based on database-derived cases, the data may contain subjective biases, though we sought to minimize this by incorporating objective index into our analysis; Third, the number of cases defined as treatment failures was relatively small, possibly due to incomplete follow-up data for these patients. Fourth, there were challenges in categorizing some subjective indicators, such as differentiating between systemic and localized aversion to cold, which may involve distinct neural pathways, thus lacking finer evaluation. Fifth, the scope of inflammatory, immunological, and imaging-related parameters included in this study was limited, hindering the model from reaching its full predictive potential.

In future studies, we plan to collect additional external validation datasets from other regions to further refine and enhance the model's performance. We also encourage other researchers to validate their own datasets using our model parameters. To address the issue of data bias, we will expand the diversity of the sample, integrate additional biomarker data, strengthen the integration of clinical information, enhance the collection of longitudinal data, utilize multimodal data, optimize data quality control, and promote interdisciplinary collaboration. Moreover, to gain a deeper understanding of the underlying reasons for treatment failure, future research will focus on detailed analysis and data mining of these cases, aiming to further optimize treatment strategies.

## 5 Conclusions

TCM is an important treatment modality for Long COVID. The XGBoost model, built on clinical data, effectively identifies Long COVID patients who are most likely to benefit from TCM treatment. Using the SHAP method, we interpreted the model and identified key features with the highest contributions, thereby developing a clinically interpretable ML model. Additionally, we constructed a nomogram to further support the application of the ML model in both prediction and interpretation, providing a more intuitive tool for clinical decision-making.

## Data Availability

The raw data supporting the conclusions of this article will be made available by the authors, without undue reservation.
